# Metabolic Acidosis Treatment as Part of a Strategy to Curb Inflammation

**DOI:** 10.1155/2013/601424

**Published:** 2013-06-06

**Authors:** Tales Rubens de Nadai, Mariane Nunes de Nadai, Agnes Afrodite Sumarelli Albuquerque, Marco Tulio Menezes de Carvalho, Andrea Carla Celotto, Paulo Roberto Barbosa Evora

**Affiliations:** Department of Surgery and Anatomy, Ribeirão Preto Faculty of Medicine, University of São Paulo, Avenida Bandeirantes 3900, 14048-São Paulo 900, 14015-120 Ribeirão Preto, SP, Brazil

## Abstract

Abnormalities in systemic acid-base balance may induce significant changes in the immune response, and they may play a significant role in the development or maintenance of immune dysfunction. Different forms of acidosis (metabolic and respiratory) and even different types of metabolic acidosis (hyperchloremic and lactic) may produce different effects on immune function. If alkalization has, or not, some effect on inflammation control is still a matter of speculation. Studies concerning these subjects are limited justifying this paper.

## 1. Introduction

Abnormalities in systemic acid-base balance may cause significant changes in the immune response. The clinical significance of these changes is not yet fully known, but its magnitude suggests that they may play a significant role in the development or maintenance of immune dysfunction. Thus, they represent attractive targets for curbing inflammation.

Metabolic acidosis is one of the most common abnormalities in patients suffering from serious diseases. There have numerous etiologies and treatment of the underlying disease is the basis of therapy. However, there is a growing evidence suggesting that acidosis itself has profound effects on the host, particularly in immune function. Given the critical importance of immune function for the outcome of the illness, there is an overriding interest in elucidating the effects of this condition.

**Figure 1:**
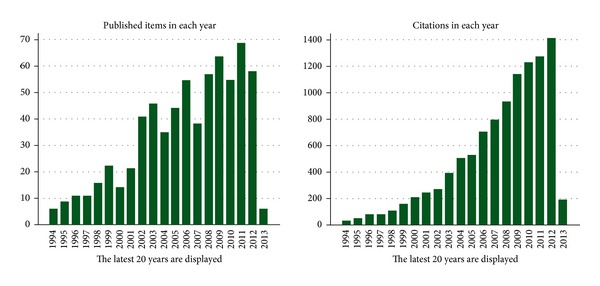
Metabolic acidosis and inflammation (Web of Science data).

In fact, recent evidence suggests that the different forms of acidosis (metabolic and respiratory) and even different types of metabolic acidosis (hyperchloremic and lactic) may produce different effects on immune function. The ways in which these effects are applied to the clinical conditions have not been determined. Therefore, since acidosis is an extremely common problem in intensive care units and that immune function is of vital importance, efforts to explain these relations are fully justified [1]. However, it is necessary to note that the publications linking acidosis with the inflammatory response are limited, and studies on the alkalosis are virtually nonexistent, justifying the current paper, at least as an open discussion (Figure 1).

## 2. Pathophysiology

The literature has reported in vitro experiments where researchers reduced intracellular pH (pHo) using different types of acids. Notably, different patterns of expression of mediator of inflammation occurred at different acids, despite the normalization of samples to the same pHo [1]. Kellum et al. [2] demonstrated that different degrees of hyperchloremic acidosis produce different effects on the release of inflammatory mediators. By using electrophoretic mobility shift, these researchers have measured the binding of NF-*κ*B DNA after exposure to different concentrations of HCl. When compared to pHo 7.4, acidosis (7.0 pHo) significantly increased the activation of NF-*κ*B induced by LPS, while more severe acidosis (6.5 pHo) attenuated the activation of NF-*κ*B, concluding that the degree of acidosis directly influences the immune function.

Unlike HCl, lactic acid has been studied in an even more limited way with even less convincing effects, and the mechanisms by which the HCl and lactate exert effects on innate immunity are unknown. Furthermore, the Kellum et al. investigation [2] evaluated the inflammatory response induced by LPS (*E. coli* toxin) associated with acidosis. The inflammatory response through quantification of inflammatory markers (NO, IL6, and IL-10 binding with DNA LPS), showed that HCl and lactic acid exhibit antagonistic responses, and an immune response increases with HCL and decreases with lactic acid. The basic conclusion is that different types of acids produced different effects on cellular immune function even when normalized to the same pH.

Evidently, the interrelationship between extracellular and intracellular pH on immune function cannot be ignored, especially in light of the numerous findings implicating a role for the Na/H exchanger prior to the activation of certain immune activities, suggesting that the Na/H exchanger is a *sine qua non *in generating a rapid intracellular alkalinization prior to the differential activation of certain immune activities [3]. However, a major criticism of the experimental studies is the strong acidification (pH 6.5–7.0), which shows more clearly the role of inflammation, and these levels of acidosis are rarely observed in the clinical setting. Moreover, it is difficult to differentiate between intracellular and extracellular acidosis, including possible “critical windows” and their relationship to inflammation.

## 3. Acid-Base Biomarkers and Inflammation

Farwell and Taylor conducted a survey about the relationship between serum acid-base status and inflammation. This study examined the relationship between serum anion gap, bicarbonate levels, and inflammatory biomarkers in healthy subjects. It was shown that a higher anion gap and a lower level of serum bicarbonate (despite being within the normal range) were associated with higher levels of several inflammatory biomarkers, including leukocyte count and levels of C-reactive protein. The cause of the observed relationship between higher serum anion gap and higher levels of inflammatory markers in this apparently healthy people is unknown. These data raise the possibility that the increased production of organic acids measured the chronic inflammatory disease, which increases the risk of coronary heart disease and cancer. More studies are necessary to determine the effectiveness of alkali supplementation on levels of inflammatory biomarkers [4].

Lactate clearance, a surrogate for the magnitude and duration of global tissue hypoxia, is used diagnostically, therapeutically, and prognostically. Nguyen and colleagues examined the association of early lactate clearance with selected inflammatory, coagulation, apoptosis response biomarkers, and organ dysfunction scores in severe sepsis and septic shock [5]. They carried out measurements of serum arterial lactate, and biomarkers (interleukin-1 receptor antagonist, interleukin-6, interleukin-8, interleukin-10, tumor necrosis factor-alpha, intercellular adhesion molecule-1, high mobility group box1, D-dimer, and caspase-3). In addition, organ dysfunction scores (Acute Physiology and Chronic Health Evaluation II, Simplified Acute Physiology Score II, Multiple Organ Dysfunction Score, and Sequential Organ Failure Assessment) were obtained in conjunction with a prospective, randomized study. Lactate clearance was defined as the percent change in lactate levels after six hours from a baseline measurement in the emergency department. The results of the statistical analysis showed that early lactate clearance as a surrogate for the resolution of global tissue hypoxia was significantly associated with decreased levels of biomarkers, improvement in organ dysfunction, and outcome in severe sepsis and septic shock. This well-designed study was selected because it illustrates the relationship between metabolic acidosis and the inflammation reaction.

## 4. Therapeutic Aspects

The various studies described above suggest that changes in systemic acid-base balance can also cause alterations in the immune response through several different ways. Thus, further investigation of these changes may lead to valuable therapeutic targets in treating some potentially serious diseases.

Acidosis and changes in intracellular and extracellular pH may influence endothelial function in different aspects. In large arteries, intracellular acidosis is associated with vasodilatation, whereas in small arteries, it leads to vasoconstriction. The pathogenic events of this response are not well known, but it was demonstrated that acidosis regulates not only the iNOS but also the eNOS [6]. Nitric oxide can contribute to the control of local blood flow during hypoxia/ischemia, which presents a close relationship with lactic acid production. This analysis suggests that the pH regulation may represent a potential therapeutic target in curbing inflammation. This new approach may attenuate endothelial dysfunction in diseases associated with acidosis [7].

Nowadays to consider what the best strategies to treat metabolic acidosis, considering the inflammatory response is an exercise in speculation and hypotheses. For example, there are advantages to seek alternative ways to treat intracellular acidosis, or just to know that it comes concomitant to the extracellular treatment? If have advantages, “resurrect” the TRIS buffer clinical use would be considered since this buffer is theoretically more effective to treat the intracellular acidosis, than ever questioned bicarbonate?

The “folk” thought that the patient died of multiple organ failure with hemogasometry normal is particularly metaphorical and capable of deep reflection. The correction of metabolic acidosis as an isolated marker needs to be abandoned and considered being an essential part of the systemic inflammatory response. Thus, administration of potent and selective NHE1 inhibitors afford protection from the whole body ischemia-reperfusion injury by attenuating myocardial dysfunction and improving organ blood flow and systemic oxygen delivery, resulting in reduced proinflammatory response [8].

## 5. Conclusion

Most often, metabolic acidosis is present in acute systemic inflammatory response in which the control of acid-base balance is part of the treatment protocol. Thus, evaluation of the role of metabolic acidosis is mandatory. One of main concerns about this paper is the difficulty to establish “what the chicken is and what the egg is.” In many cases, acute acidosis is secondary to, for example, circulatory shock, and one could wonder whether, under those conditions, the circulatory shock causes the inflammatory response or the acidosis related to the shock. The same reasoning can be followed in patients who develop respiratory acidosis due to ARDS or COPD, as the lung disease by itself will induce an inflammatory response. Perhaps the most unequivocal data providing evidence for an impairment of the immune response appear from the clinical studies of the organic acidosis and ketoacidosis. In general, the clinical acidemias are accompanied by immunodeficiency, including a reduction in white cell numbers, gamma globulins, mitogenic responses, a diminution of the inflammatory response, and delayed phagocytosis. In many cases, the immunodeficiency is reversed on the correction of the acidosis. Despite the valuable research carried out to date, a lack in the appreciation of extracellular acid-base effects on a wide range of other immune activities exists [3]. Therefore, the situations in which acidosis remains steadily, as chronic renal failure, would be more suitable for the evaluation of the treatment to curb the spread of the inflammatory process.

It should be emphasized that the metabolic acidosis is common in critically ill patients and its presence can have a detrimental effect on clinical outcome. The administration of base, a common therapeutic maneuver, does not appreciably improve clinical outcome, even when acidosis is improved [9]. Is it better to consider “body acid-base imbalance” than “blood acid-base imbalance”?

*Metabolic Acidosis and Inflammatory Response Key Points*

- Metabolic acidosis is one of the most common abnormalities in patients suffering from serious diseases, and there is a growing evidence suggesting that acidosis itself has profound effects on the host, particularly in immune function.
- Recent evidence suggests that the different forms of acidosis (metabolic and respiratory) and even different types of metabolic acidosis (hyperchloremic and lactic) may produce different effects on immune function.
- Publications linking acidosis with the inflammatory response are limited, and a major criticism of the experimental studies is the strong acidification (pH 6.5–7.0), which shows more clearly the role of inflammation, and these levels of acidosis are rarely observed in the clinical setting.
- Anion gap, bicarbonate, and lactate are possible biomarkers of the inflammation response.
- Perhaps the most unequivocal data providing evidence of the immune response impairment emerge from the clinical studies of the organic acidosis and ketoacidosis. In general, the clinical acidemias are accompanied by immunodeficiency, including a reduction in white cell numbers, gamma globulins, and mitogenic responses, a diminution of the inflammatory response.
- Nowadays, to consider what the best strategies to treat metabolic acidosis, considering the inflammatory response is an exercise in speculation and hypotheses.
- The administration of selective inhibitors of NHE1 minimizes the degree of cellular injury and improves survival.
- The correction of metabolic acidosis as an isolated marker needs to be abandoned and considered as being an essential part of the systemic inflammatory response. Is it better to consider “body acid-base-imbalance” than “blood acid-base-imbalance”?
